# Implications of the choice of distance-based measures in assessing and investigating tumble turn performance

**DOI:** 10.3389/fspor.2022.958548

**Published:** 2022-09-22

**Authors:** Paul Koster, Wouter Arnoldus, Sina David, Sander Schreven, Peter J. Beek

**Affiliations:** ^1^InnoSportLab de Tongelreep, Eindhoven, Netherlands; ^2^Department of Human Movement Sciences, Vrije Universiteit Amsterdam, Amsterdam Movement Sciences, Amsterdam, Netherlands

**Keywords:** swimming, flip turn, performance measures, turning technique, sex, performance level

## Abstract

Although the tumble turn in swimming has been studied extensively, no consensus exists about which measure is best suited to capture its performance. The aim of this study was to better understand the implications of choosing a particular distance-based performance measure for assessing and investigating tumble turn performance in freestyle swimming. To this end, a large set of retrospective turn data consisting of 2,813 turns performed by 160 swimmers was analyzed statistically in three steps. First, a mixed-effects model was derived for the entire data set, which showed that both performance level and sex had clear effects on the distance-based performance measures and performance determining variables studied in the literature. Second, repeated measures correlations were calculated for the entire data set and four performance level- and sex-based subgroups to determine the level of association between the performance measures. This analysis revealed that the performance measures were strongly correlated (*r* > 0.84 and *p* < 0.05 for all possible pairs), largely independent of performance level and sex. This finding implies that the choice of performance measure is not very critical when one is interested solely in the overall performance. In the third and last step, mixed-effects models were derived for the performance measures of interest to establish the importance of different turn-related actions for each measure, again for both the entire data set and the four subgroups separately. The results of this analysis revealed that performance measures with short(er) distances are more sensitive to changes in the adaptation time and reflect the wall contact time better than performance measures with long(er) distances, which in contrast are more useful if the focus is on the approach speed prior to the turn. In this final analysis, various effects of performance level and sex were found on the technical execution of the tumble turn.

## Introduction

Turning is an important component of competitive swimming, which determines a substantial portion of the overall swim performance. Research has shown that the accumulated duration of turns amounts to at least 19% of the total race time in long-course races and up to 44% in short-course races (Veiga et al., 2013; Morais et al., 2019a,b; Born et al., 2021). However, no consensus exists in the literature about the most suitable operational measure for capturing the time associated with performing the most common turn in swimming, the freestyle tumble turn.

In the literature, two types of definitions for turning performance are found: one based on the time that elapses between the submerging of the head at turn initiation and its resurfacing after the turn, thus covering a variable distance covered within this interval, and one based on the time that elapses between the head (or another body part) crossing predefined fixed distances toward and away from the wall. The limitation of the first definition is that a lower score on this performance measure (a shorter time) does not necessarily indicate a superior turn performance compared to a higher score (a longer time). This is due to the velocity profile of the swimmer after the push-off from the wall and the subsequent underwater phase. The velocity after the push-off from the wall is higher than the free-swimming speed and decreases over time due to drag until the speed is equal to the free-swimming speed, at which the swimmer ideally transfers to free swimming (Shimadzu et al., 2008). This moment can be postponed by extending the underwater phase [e.g., by making (more) undulatory kicks], however on the first, action-based definition, the performance score becomes lower (better) even if the swimmer resurfaces before the optimal point is reached. The sooner the swimmer resurfaces, the lower (better) the score on this performance measure will be. However, this lower score will typically not translate into a better overall swim performance.

Although the use of fixed distances overcomes this problem, there is quite some variation in the literature regarding the reference distances that are used to measure turning performance (Silveira et al., 2011). This variation exists because there are no distances that naturally limit the turn, except that, according to race regulations, the head must resurface before the 15-m mark after the turn for all swim strokes except the breaststroke. Obviously, the resulting variation in reference distances limits the comparison of results across studies. The performance measures that have been used in the literature are the 5 m round trip time (5mRTT), i.e., the time that elapses between the swimmer crossing the 5 m line away from the wall when approaching it (5 m-in) and crossing it again when moving out of the wall (5 m-out) (Blanksby et al., 1996, 1998; Cossor et al., 1999; Clothier, 2004; Mosavi et al., 2012; Pereira et al., 2015; Smithdorf, 2018; Nicol et al., 2019), the 2.5mRTT (Toshiaki et al., 2001), the 3mRTT (Puel et al., 2010a,b, 2012), the 7.5mRTT (Mason and Cossor, 2001; Tourny-Chollet et al., 2002; Shahbazi et al., 2007; Bahadoran et al., 2012; Veiga et al., 2013; Skyriene et al., 2017), the 5 m-in to 7.5 m-out time (Nicol et al., 2019), the 5 m-in to 10 m-out time (Webster et al., 2011; Nicol et al., 2019) and the 5 m-in to 15 m-out (McCabe et al., 2012; Suito et al., 2015; Morais et al., 2019a,b; Nicol et al., 2019; Marinho et al., 2020) time.

Based on empirical observations, Silveira concluded that the 5mRTT constitutes the best measure to describe turning performance in sub-elite swimmers because it included all turn-related actions and had the lowest percentage of free-swimming compared to other fixed-distance performance measures (Silveira et al., 2011). However, it remains unclear whether the turns of swimmers of different performance levels and/or sex, who might submerge or resurface earlier or later than 5 m from the wall, are also optimally reflected by the 5mRTT.

It is important to note in this context that the freestyle tumble turn is a highly complex skill, which involves many different actions that are performed sequentially in rapid succession. For instance, it takes about 1.1 s from turn initiation to the feet leaving the wall. Within this short time, the swimmer slows down, reverses position, and generates as much speed as possible in the opposite direction by applying a high push-off force to the wall. Immediately thereafter an underwater phase follows involving both passive gliding and active propulsion actions. Hence, in general, it is not realistic to assume that a performance measure that ignores speed and distance and is based on time alone can fully capture the freestyle tumble turn performance with all its performance determining factors.

Nonetheless, time-based performance measures can be used not only to assess the turn performance by itself but also to gain insight into the determinants of turn performance that might be improved through training. Based on previous analyses of the tumble turn, the technical variables that might be relevant in this regard include the approach speed toward the wall (Lyttle and Mason, 1997), the horizontal distance at which the turn is initiated (Blanksby et al., 1996; Lyttle and Mason, 1997), the adaptation time [from head submersion until the feet touching the wall (Lyttle and Mason, 1997; Maglischo, 2003)], the wall contact time (Blanksby et al., 2004; Pereira et al., 2006; Araujo et al., 2010), the push-off angle, the deepest point reached by the hips during underwater swimming (Lyttle et al., 1998), the horizontal distance from the wall at first downbeat (Lyttle et al., 2000), and the break-out distance (i.e., the horizontal distance from the wall at which the head breaks the water surface (Mason and Cossor, 2001).

To our knowledge, no other attempts besides Silveira (Silveira et al., 2011) have been made to examine the relative merits of the turning performance measures used in the literature, nor the information contained in them. The aim of the present study was to fill this lacuna by analyzing a large set of retrospective turn data that were collected from 160 elite and sub-elite swimmers of both sexes during regular training sessions. To this end, three statistical analyses were performed: (1) a foundational analysis to examine the impact of performance level and sex on the performance measures and performance determining variables that have been studied in the literature, (2) a coarse-grained analysis of the correlation strengths between the performance measures of interest, and (3) a fine-grained analysis of the degree to which the aforementioned technical variables account for the variation in each of the performance measures of interest. For all three analyses, we had specific expectations based on a combination of common sense and previous findings.

Considering the well-documented performance differences between male and female swimmers and between elite and sub-elite swimmers (Arellano et al., 1994; McCabe et al., 2012; Veiga and Roig, 2016; Morais et al., 2019b; Nicol et al., 2019; Marinho et al., 2020), we expected both performance level and sex to have a significant effect on the performance measures and performance determining variables of interest. We conducted this preliminary foundational analysis to determine how to treat the effects of performance level and sex in the subsequent statistical analyses (see section Materials and methods for details).

The coarse-grained analysis was motivated by the recognition that the information contained in the different performance measures is overlapping due to their overlapping fixed in and out distances. As a result, the performance measures will be correlated, which raises the question of how critical the choice of a particular performance measure is relative to other possible choices. After all, the higher the association between the performance measures, the less critical the choice of performance measure becomes. Based on logical grounds, we expected the association between measures to be high and largely independent of the performance level and sex of the participants. If so, this would render the choice of performance measure less critical from a statistical point of view.

The fine-grained analysis is based on the recognition that the tumble turn is a highly complex skill. As a result, the choice of a particular performance measure may reflect some turn-related actions more prominently and other turn-related actions less prominently, if at all. In other words, the information contained in a particular performance measure is likely to be a function of its definition. To select a suitable performance measure for answering a certain scientific or practical question, it is necessary to understand which information is contained in the set of (currently) available performance measures. This can be accomplished by deriving linear mixed-effects models to determine which specific turn-related actions account for (most of) the variance of each performance measure.

Based on logical grounds, we expected the technical variables associated with wall contact, notably the adaptation time and wall contact time, to account for more of the variance of the performance measures with a short(er) distance covered (that is, with fixed in and out distances close to the wall) than for performance measures with a long(er) distance covered (that is, with fixed in and out distances far from the wall). Conversely, we expected technical variables associated with the underwater phase, notably the push-off angle, the deepest point of the hip reached underwater and the break-out distance, to account for more of the variance of performance measures with a long(er) distance than performance measures with a short(er) distance covered.

## Materials and methods

### Participants

For this study, extensive retrospective freestyle turn data from 160 swimmers (85 male, 75 female) were analyzed. The participants, or their legal guardians in case they were 16 years of age or younger, signed an informed consent form stating that their turn data would be used anonymously for the purpose of scientific research. FINA points were determined to assess the performance level of the swimmers. To this end, the race times on any freestyle event (long course or short course) swum during the period from 2010 to 2021 in which the turn data were collected, were converted to FINA points based on the FINA point score of 2021 (FINA World Championships, 2022). The highest FINA point score of all these races was used to quantify the performance level of each swimmer. The 50 m long events were excluded from this procedure as they contain no turn.

The participants were categorized as either elite or sub-elite swimmers, depending on their FINA point score, which enabled making statistical comparisons based on performance level. Swimmers were considered elite swimmers if they had 860 FINA points or more. The 860 FINA points cut-off for elite swimmers was based on the average FINA point score corresponding to the FINA A qualification time for the freestyle events of the World Championships 2022 (Bouget, 2008).

### Data acquisition

The tumble turn data for this study were derived from video footage recorded between 2010 and 2022 with a video system in the training pool of InnoSportLab de Tongelreep in Eindhoven. Before the measurements, a marker was placed on the swimmer's trochanter major to capture the position of the hip to derive the technical variables of interest (see below for further details).

The fixed video system in the training pool of De Tongelreep consists of four cameras (scA 1400-30gc, 50 Hz, Basler, Ahrensburg, Germany) that are embedded in the pool's lateral sidewall (at respectively, 2, 5, 10, and 15 m from the turn wall at a depth of 0.55 m below the water surface). The video data from the cameras were acquired using the software package Streampix 7 (Norpix, Montreal, Canada, 2016). The cameras were synchronized by means of an external trigger pulse generator (NI-DAQmx Pulse Generator). During the trials, the swimmers began swimming about 15 m from the wall (before the turn), sprinted toward the wall, turned and sprinted back until they were positioned well beyond the 15-m mark with their hip. All trials were recorded during regular training sessions and performed at maximal or race pace effort to mimic competitive races. Whether or not this was actually accomplished was not considered a major concern since having considerable variation in tumble turn performances is beneficial for the to-be-performed statistical analyses.

All video recordings were analyzed manually using a custom-made software package called Turnanalyzer (Escrito IT, Eindhoven, The Netherlands). The intrinsic parameters of the cameras were determined with the Camera Calibration Toolbox in Matlab (R Core Team, 2022) using image data of a checkerboard from various positions. The extrinsic calibration parameters were obtained by making use of control points at known positions in the pool. The intrinsic and extrinsic camera parameters were combined to reconstruct pixel data in the field of view of the cameras to 2D real-world coordinates on a sagittal plane positioned parallel to the sidewall at a distance of 3.6 m into the pool. The calibration error (root mean squared error) of the cameras was between 0.40 and 0.52 pixels for the cameras positioned at 2, 10, and 15 m, respectively, and 1.82 pixels for the camera positioned at 5 m, which was thus a factor less accurate than the other cameras. The technical variables were then manually extracted from the video and the resulting data were stored in a database.

### Data preparation

**Figure 1:**
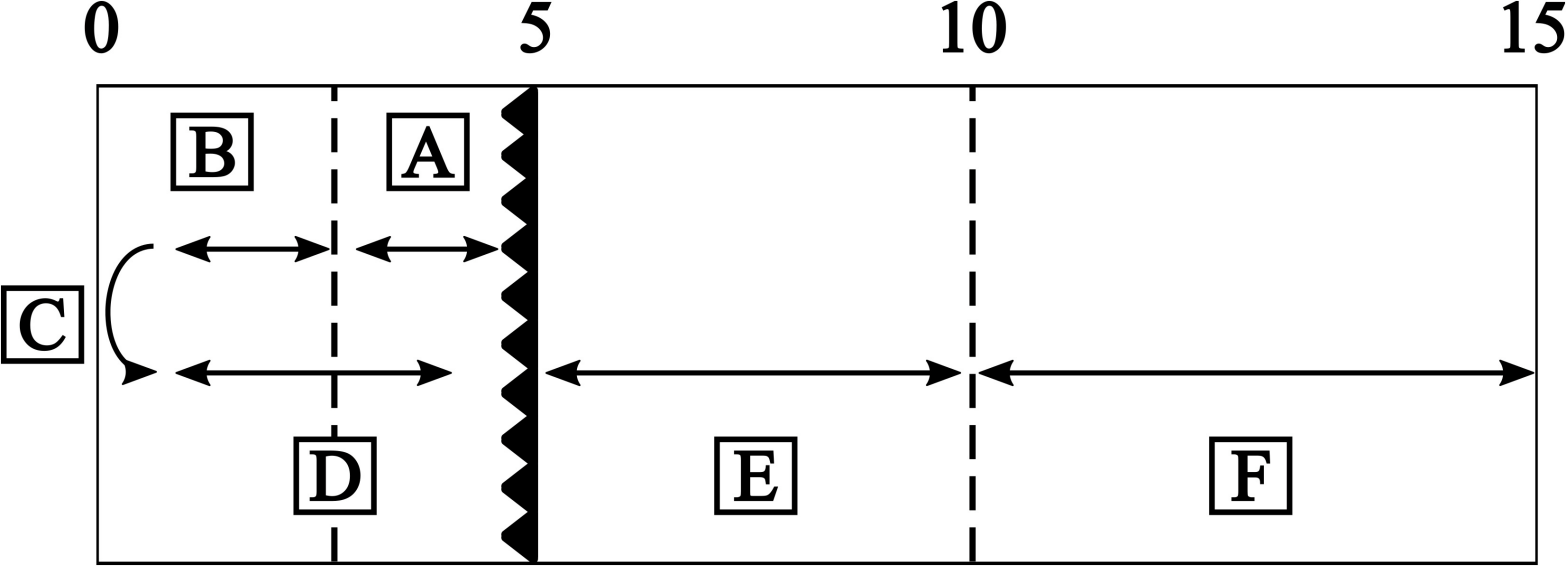
Graphical representation of the temporal variables and distances used for calculating the performance measures and the technical variables of interest. A: from 5 m-in until 3 m-in to the wall, B: from 3 m-in to the wall until head down before the turn, C: duration of the tumble turn and push-off (starting with the head underwater until the feet lose contact with the wall), D: the feet having lost contact with the wall until the hip crosses the 5 m mark, E: from 5 m-out of the wall until 10 m-out of the wall and F: 10 m-out of the wall until 15 m-out of the wall.

All front crawl turn data of the included swimmers were extracted from the database and filtered for trials that contained all of the temporal performance measures depicted in Figure 1: from 5 m-in to 3 m-in to the wall (A), from 3 m-in to the wall until the moment the head is completely submerged before the turn (B), the time taken for the tumble turn and push-off [from head under the water surface until the feet losing contact with the wall (C)], the feet losing contact with the wall until hip at 5 m (D), from 5 m-out of the wall until 10 m-out of the wall (E) and 10 m-out of the wall to 15 m-out of the wall (F). The hip was used as the reference point for establishing the time at which the various spatial variables of interest were determined. In the protocol used in De Tongelreep for the measurement and analysis of swimming movements, the hip is routinely chosen for this purpose, because it is always in the field of view of the camera(s), regardless of the swimming stroke used (as opposed to the head, which is continuously visible in freestyle swimming, but not, for example, in the breaststroke and butterfly).

Based on the temporal variables used in the literature, the following temporal performance measures were determined for each turn: 5 m-in to 5 m-out, 3 m-in to 5 m-out, 3 m-in to 10 m-out, 5 m-in to 10 m-out, 3 m-in to 15 m-out and 5 m-in to 15 m-out.

Only swimmers with a minimum of five turns in the database were included in the statistical analyses to limit the impact of swimmers with only a small number of turns in the database satisfying the selection criteria. Furthermore, trials were excluded if they did not contain data on all of the eight technical variables of interest, as defined operationally in Table 1.

**Table 1:** Operational definition of the variables of interest.

Speed-in (m/s)	The average approach speed between the 5 and 3 m mark before the turn.
Initiation distance (m)	The horizontal distance of the hip to the wall at the moment the head of the swimmer submerges.
Adaptation time (s)	The time needed to bring the feet to the wall measured from the moment the head completely submerges until the first wall contact.
Wall contact time (WCT) (s)	The time between the first moment the feet are touching the wall until the last moment they are touching the wall.
Push-off angle (**°**)	The angle in the sagittal plane of the lane between a horizontal line and a line from the toes to fingertips at the moment of last wall contact. An angle of 0 degrees corresponds to a horizontal orientation of the body.
Deepest point (m)	The deepest point of the hips during the underwater swimming phase.
Distance of first kick (m)	The horizontal distance of the hip from the wall at the first downbeat.
Break-out distance (m)	The horizontal distance of the hip from the wall at the moment of resurfacing.

### Statistical analysis

**Figure 2:**
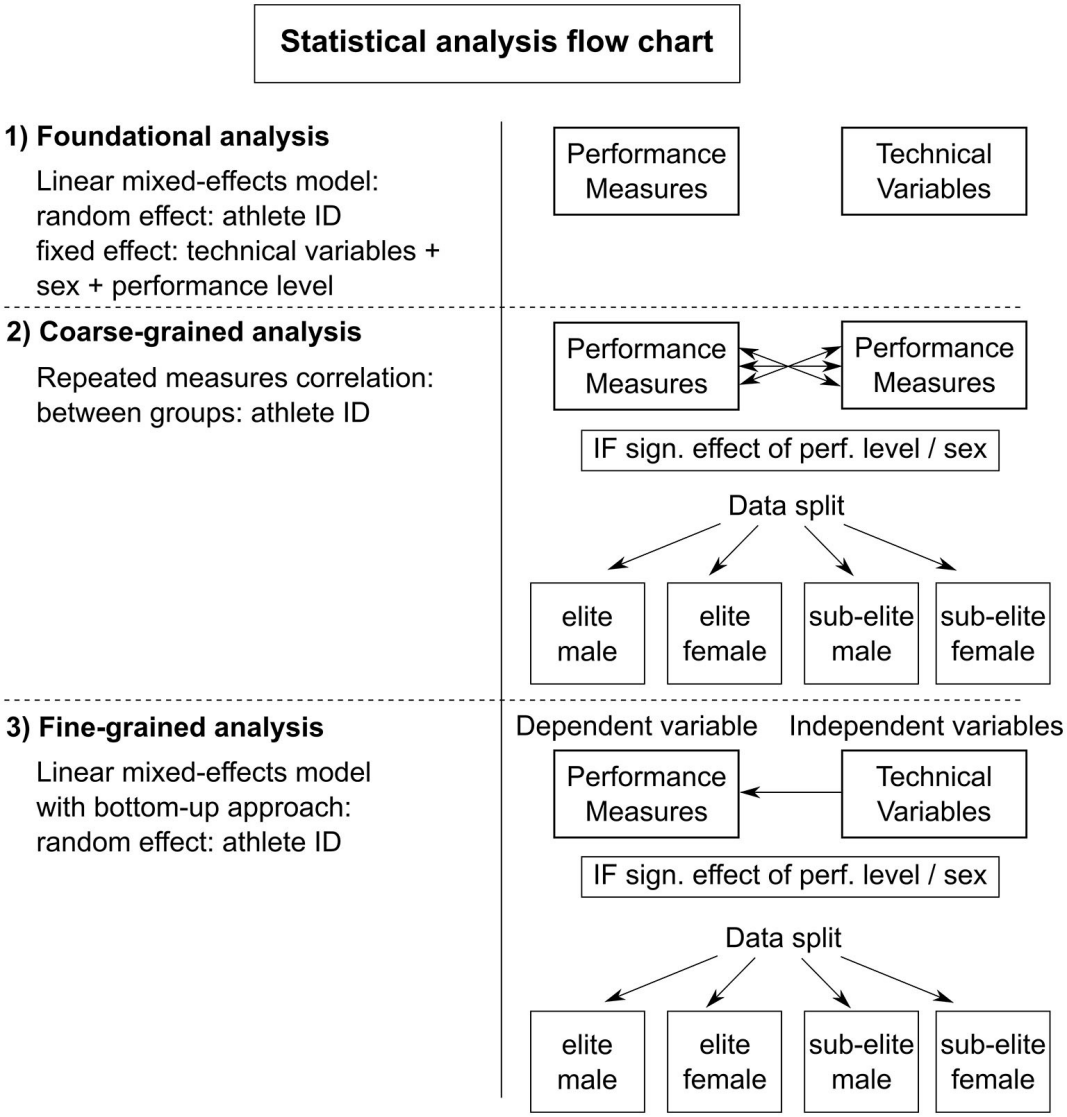
Flow chart of the performed statistical analysis. 1) Foundational analysis to investigate the effect of performance level and sex on the performance measures and technical variables. 2) Coarse-grained analysis to determine the level of association between the performance measures. 3) Fine-grained analysis to examine the relationship between each performance measure and the selected technical variables.

All statistical analyses were performed in R (Bates et al., 2014) using RStudio 4.2.0 (RStudio, Boston, Massachusetts, USA). In particular, the following modules were used: lme4 (Wickham et al., 2022), readxl (Barton, 2022), MuMIn (Long, 2022) and jtools (Bakdash and Marusich, 2022) and rmcorr (Bakdash and Marusich, 2017; Schweinberger, 2022). The statistical analysis of the data consisted of (1) a foundational analysis, followed by (2) a coarse-grained analysis, and (3) a fine-grained analysis (see Figure 2). For all statistical tests, an α-level of 0.05 was assumed.

### Foundational analysis

First, the effects of performance level and sex on the performance measures and technical variables were assessed in a foundational analysis of the entire data set by deriving linear mixed-effect models for each of the six performance measures and the eight technical variables separately. In this analysis, the technical variables, performance level and sex were defined as fixed effects and athlete ID as a random effect (Figure 2).

As indicated, the main aim of this analysis was to determine how to treat the effects of performance level and sex in the subsequent statistical analyses. In particular, if a significant main effect of either performance level or sex would be found for (the majority of) the performance measures, the data set would be split into two subgroups (male and female, or elite and sub-elite, depending on the nature of the effect). Similarly, if main effects of both performance level and sex would be found for (the majority of) the performance measures, the data would be split into four subgroups (i.e., elite male, elite female, sub-elite male and sub-elite female, Figure 2). Splitting the data accordingly into subgroups may provide insight into possible group-specific differences in the performance measures and different turn strategies with regard to the technical, performance determining variables of interest. In this regard, the described statistical analysis was foundational for the subsequent coarse-grained and fine-grained analysis of the entire data set, and whether or not they would be complemented by group-specific analyses (Figure 2).

### Coarse-grained analysis

To investigate the level of association between the different performance measures, repeated measures correlations were calculated for all possible pairs of performance measures, either for the complete data set alone or also for specific subgroups, depending on the results of the foundational analysis. This method is more suitable than Pearson's correlation analysis to deal with the repeated measures design of this data set and the unequal number of observations per individual. In particular, it is not biased by the mixing of intra- and inter-individual variance, where Pearson's correlation analysis would be (Schweinberger, 2022). The lower the repeated measures correlation coefficient, the more important the choice of one performance measure in favor of the other.

### Fine-grained analysis

In the next, in-depth analysis, linear mixed-effect models with a bottom-up procedure (Lyttle et al., 1999) were constructed to determine the relationship between each performance measure and the eight derived technical variables as independent predictors (Figure 2). This was done to estimate the sensitivity of each performance measure with regard to the technical variables (Table 1) that were identified in previous research as potentially important determinants of turn performance (Blanksby et al., 1996, 2004; Lyttle and Mason, 1997; Welham and Thompson, 1997; Mason and Cossor, 2001; Maglischo, 2003; Pereira et al., 2006; Araujo et al., 2010). Again, this was either accomplished for the complete data set alone or also for (two or four) specific subgroups, depending on the result of the foundational analysis (Figure 2).

As a first pass on the data, boxplots and histograms were made and inspected for all independent variables to identify outliers amongst the independent predictors. Following this, collinearity was assessed by calculating the repeated measures correlation coefficients between all independent variables. In case there was a strong repeated measures correlation (***r*** > 0.7) between two independent variables, the one with the highest absolute correlation coefficient with the performance measure was preserved as a predictor in the model while the other one was removed.

Finally, a bottom-up procedure (Lyttle et al., 1999) was used to determine which (combination of) independent variables provided the best fit for the model, starting with a model that only contained athlete ID as a random effect, due to the repeated measures design of the data. In each iteration round, a variable was added to the linear mixed-effects model and the new model was compared to the previous one using the likelihood ratio test (Nakagawa and Schielzeth, 2013). If the model was not significantly different from the previous one, the latest added variable was removed from the model. This procedure was continued until the model only contained variables that improved the model's fit. To be able to compare the contributions of the independent variables within and between the models, the same models were constructed with the standardized dependent and independent variables, thereby removing the effect of the scale at which they were measured. The following equation was used to standardize the variables:


(1) $$\begin{array}{l} z_{i}=\frac{x_{i}-\mu }{sd_{X}} \end{array}$$

where *z*_*i*_ represents the standardized value, *x*_*i*_ represents the unstandardized value, μ represents the sample mean of the variable and *sd* is the sample standard deviation of the variable. Finally, the *R*^2^ values of the linear mixed-effects models were determined using the method of Nakagawa and Schielzet (Lyttle et al., 1998).

## Results

### Foundational analysis

After application of the inclusion criterion to all turns available in the database, a total of 2,813 turns from 160 individual swimmers remained, out of which 1,154 turns were performed by 38 elite male and female swimmers combined (Table 2). The means and standard deviations of the performance measures and technical variables for both performance level and sex are displayed in Table 2. As expected, there was a significant main effect for both performance level and sex for all performance measures, in the absence of a significant sex by performance level interaction. The male swimmers were significantly faster than the female swimmers on all performance measures in both the elite and sub-elite subgroups. Furthermore, elite swimmers were significantly faster than the sub-elite swimmers on all performance measures in both the male and female subgroups. The results for the technical variables of interest are presented in Table 2. Since both performance level and sex had a significant main effect on all performance measures, both the course-grained and fine-grained analyses were performed on the entire data set and the elite male, elite female, sub-elite male and sub-elite female subgroups separately.

**Table 2:** Descriptive statistics and means and standard deviations of performance measures and technical variables for the elite male and female and sub-elite male and female subgroups separately.

**Descriptive statistics**					
	**Sex**	**Elite**	**Sub-elite**		
Number of swimmers	Male	23	62		
	Female	15	60		
Number of trials	Male	600	670		
	Female	554	989		
Age (years)	Male	21.5 ± 3.2	18.9 ± 3.6		
	Female	23.7 ± 5.4	19.5 ± 4.3		
FINA points	Male	899 ± 22	684 ± 131		
	Female	935 ± 53	750 ± 87		
**Performance measures**	**Sex**	**Elite**	**Sub-elite**	***F*** **-statistic (sex)**	***F*** **-statistic (performance level)**
3 m in−5 m out	Male	3.54 ± 0.30	4.00 ± 0.47	*F*_(1,151.1)_ = 43.39	*F*_(1,151.1)_ = 37.43
	Female	3.88 ± 0.25	4.36 ± 0.42	*P* < 0.001, $\eta_{\text{p}}^{2}$ = 0.22	*P* < 0.001, $\eta_{\text{p}}^{2}$ = 0.19
5 m in−5 m out	Male	4.62 ± 0.40	5.14 ± 0.57	*F*_(1,155.47)_ = 46.01	*F*_(1,155.47)_ = 35.03
	Female	5.02 ± 0.30	5.60 ± 0.52	*P* < 0.001, $\eta_{\text{p}}^{2}$ = 0.23	*P* < 0.001, $\eta_{\text{p}}^{2}$ = 0.18
3 m in−10 m out	Male	6.37 ± 0.49	7.11 ± 0.80	*F*_(1,155.35)_ = 37.66	*F*_(1,155.35)_ = 33.65
	Female	6.91 ± 0.38	7.71 ± 0.72	*P* < 0.001, $\eta_{\text{p}}^{2}$ = 0.20	*P* < 0.001, $\eta_{\text{p}}^{2}$ = 0.18
5 m in−10 m out	Male	7.45 ± 0.58	8.25 ± 0.90	*F*_(1,155.55)_ = 39.91	*F*_(1,155.55)_ = 32.74
	Female	8.05 ± 0.44	8.96 ± 0.82	*P* < 0.001, $\eta_{\text{p}}^{2}$ = 0.20	*P* < 0.001, $\eta_{\text{p}}^{2}$ = 0.17
3 m in−15 m out	Male	9.20 ± 0.71	10.18 ± 1.11	*F*_(1,155, 75)_ = 38.19	*F*_(1,155.75)_ = 31.47
	Female	9.95 ± 0.54	11.04 ± 1.01	*P* < 0.001, $\eta_{\text{p}}^{2}$ = 0.20	*P* < 0.001, $\eta_{\text{p}}^{2}$ = 0.17
5 m in−15 m out	Male	10.28 ± 0.80	11.33 ± 1.21	*F*_(1,155.88)_ = 39.72	*F*_(1,155.88)_ = 30.91
	Female	11.10 ± 0.60	12.28 ± 1.11	*P* < 0.001, $\eta_{\text{p}}^{2}$ = 0.20	*P* < 0.001, $\eta_{\text{p}}^{2}$ = 0.17
**Technical variables**	**Sex**	**Elite**	**Sub-elite**	***F*** **-statistic (sex)**	***F*** **-statistic (performance level)**
Speed-in (m/s)	Male	1.88 ± 0.18	1.77 ± 0.17	*F*_(1,153.99)_ = 52.25	*F*_(1,153.99)_ = 21.21
	Female	1.75 ± 0.12	1.62 ± 0.15	*P* < 0.001, $\eta_{\text{p}}^{2}$ = 0.25	*P* < 0.001, $\eta_{\text{p}}^{2}$ = 0.12
Initiation dist. (m)	Male	1.98 ± 0.24	1.82 ± 0.18	*F*_(1,152.26)_ = 45.81	*F*_(1,152.26)_ = 11.2
	Female	1.76 ± 0.14	1.71 ± 0.17	*P* < 0.001, $\eta_{\text{p}}^{2}$ = 0.27	*P* = 0.001, $\eta_{\text{p}}^{2}$ = 0.08
Adaptation time (s)	Male	0.85 ± 0.13	0.87 ± 0.12	*F*_(1,143.88)_ = 0.02	*F*_(1,143.88)_ = 5.33
	Female	0.83 ± 0.08	0.89 ± 0.12	*P* = 0.88, $\eta_{\text{p}}^{2}$ < 0.01	*P* = 0.02, $\eta_{\text{p}}^{2}$ = 0.04
WCT (s)	Male	0.25 ± 0.07	0.30 ± 0.10	*F*_(1,137.89)_ = 0.22	*F*_(1,137.89)_ = 8.97
	Female	0.26 ± 0.06	0.30 ± 0.11	*P* = 0.64, $\eta_{\text{p}}^{2}$ < 0.01	*P* = 0.003, $\eta_{\text{p}}^{2}$ = 0.06
Push-off angle (°)	Male	7.59 ± 3.42	7.73 ± 3.88	*F*_(1,144.86)_ = 4.41	*F*_(1,144.86)_ = 0.68
	Female	8.19 ± 3.65	8.36 ± 3.73	*P* = 0.04, $\eta_{\text{p}}^{2}$ = 0.03	*P* = 0.41, $\eta_{\text{p}}^{2}$ < 0.01
dist. First kick (m)	Male	2.72 ± 0.53	1.82 ± 0.40	*F*_(1,141.78)_ = 17.82	*F*_(1,147.78)_ = 0.03
	Female	2.57 ± 0.44	2.55 ± 0.44	*P* < 0.001, $\eta_{\text{p}}^{2}$ = 0.11	*P* = 0.87, $\eta_{\text{p}}^{2}$ < 0.01
Deepest point (m)	Male	0.76 ± 0.15	0.70 ± 0.16	*F*_(1,142.2)_ = 3.07	*F*_(1,142.2)_ = 5.45
	Female	0.73 ± 0.15	0.69 ± 0.15	*P* = 0.08, $\eta_{\text{p}}^{2}$ = 0.02	*P* = 0.02, $\eta_{\text{p}}^{2}$ = 0.04
Break-out dist. (m)	Male	7.97 ± 1.90	7.24 ± 1.60	*F*_(1,150.14)_ = 7.18	*F*_(1,150.14)_ = 7.68
	Female	7.92 ± 1.63	6.74 ± 1.37	*P* < 0.001, $\eta_{\text{p}}^{2}$ = 0.05	*P* < 0.001, $\eta_{\text{p}}^{2}$ = 0.05

Furthermore, the main and interaction effects of the foundational analysis.

### Coarse-grained analysis

As can be appreciated from the repeated measures correlation matrices presented in Table 3, all performance measures were strongly (*r* > 0.8) and significantly (*p* < 0.001) correlated. For the sake of clarity, the repeated measures correlations were presented for the entire data set only, since the results for the four subgroups, which are presented in Table 3, only showed marginal differences. The highest correlation of 0.99 was between the 3 m-in−15 m-out and 5 m-in−15 m-out and the lowest correlation of 0.84 was between the 3 m-in−5 m-out and 5 m-in−15 m-out. As expected, the greater the overlap in distance between the performance measures, the higher the degree of association.

**Table 3:** The repeated measures correlation coefficient matrix between all possible combinations of performance measures for the entire data set and the elite male and female, and sub-elite male and female swimmers separately.

**Repeated measures correlation**											
3 m in−5 m out	0.92	0.85	0.96	0.90	0.84						
0.92	3 m in−10 m out	0.94	0.91	0.98	0.94						
0.85	0.94	3 m in−15 m out	0.85	0.93	0.99						
0.96	0.91	0.85	5 m in−5 m out	0.94	0.88						
0.90	0.98	0.93	0.94	5 m in−10 m out	0.95						
0.84	0.94	0.99	0.88	0.95	5 m in−15 m out						
**Repeated measures correlation elite male**						**Repeated measures correlation elite female**					
3 m in−5 m out	0.92	0.85	0.92	0.89	0.84	3 m in−5 m out	0.95	0.85	0.97	0.94	0.86
0.92	3 m in−10 m out	0.93	0.88	0.97	0.92	0.95	3 m in−10 m out	0.91	0.94	0.99	0.92
0.85	0.93	3 m in−15 m out	0.82	0.90	0.98	0.85	0.91	3 m in−15 m out	0.85	0.91	0.99
0.92	0.88	0.82	5 m in−5 m out	0.95	0.88	0.97	0.94	0.85	5 m in−5 m out	0.97	0.89
0.89	0.97	0.90	0.95	5 m in−10 m out	0.95	0.94	0.99	0.91	0.97	5 m in−10 m out	0.93
0.84	0.92	0.98	0.88	0.95	5 m in−15 m out	0.86	0.92	0.99	0.89	0.93	5 m in−15m out
**Repeated measures correlation sub-elite male**						**Repeated measures correlation sub-elite female**					
3 m in−5 m out	0.85	0.79	0.97	0.84	0.79	3 m in−5 m out	0.94	0.87	0.96	0.91	0.86
0.85	3 m in−10 m out	0.95	0.85	0.99	0.94	0.94	3 m in−10 m out	0.95	0.93	0.98	0.95
0.79	0.95	3 m in−15 m out	0.81	0.95	0.99	0.87	0.95	3 m in−15 m out	0.88	0.94	0.99
0.97	0.85	0.81	5 m in−5 m out	0.88	0.84	0.96	0.93	0.88	5 m in−5 m out	0.95	0.90
0.84	0.99	0.95	0.88	5 m in−10 m out	0.96	0.91	0.98	0.94	0.95	5 m in−10 m out	0.96
0.79	0.94	0.99	0.84	0.96	5 m in−15 m out	0.86	0.95	0.99	0.90	0.96	5 m in−15 m out
0.79	0.95	3 m in−15 m out	0.81	0.95	0.99	0.87	0.95	3 m in−15 m out	0.88	0.94	0.99
0.97	0.85	0.81	5 m in−5 m out	0.88	0.84	0.96	0.93	0.88	5 m in−5 m out	0.95	0.90
0.84	0.99	0.95	0.88	5 m in−10 m out	0.96	0.91	0.98	0.94	0.95	5 m in−10 m out	0.96
0.79	0.94	0.99	0.84	0.96	5 m in−15 m out	0.86	0.95	0.99	0.90	0.96	5 m in−15 m out

All correlation coefficients were significant (P < 0.001).

### Fine-grained analysis

The boxplots and histograms of the technical variables revealed no outliers. According to the procedure described in the Materials and methods section, the horizontal initiation distance was excluded from the model because it was highly correlated with the adaptation time (*r* = 0.73), indicating collinearity between these variables. Additionally, the absolute repeated measures correlation coefficient with the performance measures was lower (e.g., *r* = 0.20 initiation distance and the 5 m-in−5 m-out time compared to *r* = 0.29 between adaptation time and 5 m-in−5 m-out time). For all six performance measures, a prediction model was derived with athlete ID as a random effect and the seven remaining technical variables as independent predictors.

All the models showed a high *R*^2^ value ranging from 70 to 91% explained variance (see Appendix A). The prediction models of the performance measures that started 5 m before the wall had a higher *R*^2^ than those starting 3 m before the wall. The bottom-up procedure resulted in the intercepts, estimates, and standardized estimates of the independent variables that are collated in Appendix A. All models for the different performance measures across all four subgroups included the speed-in, adaptation time, WCT and break-out distance. Although the break-out distance improved the models' fit significantly, the overall contribution to all models turned out minimal. The estimates of adaptation time and WCT had a positive sign in the model, indicating that an increase in these independent variables was associated with an increased turning time. The opposite was true for the estimates of speed-in and break-out distance, which had a negative sign, indicating that an increase was associated with a faster turn. The distance of the first kick, push-off angle and deepest point in the underwater trajectory were included only in some models. If included, the push-off angle and deepest point had a positive sign, implying slower turn times with greater values.

**Figure 3:**
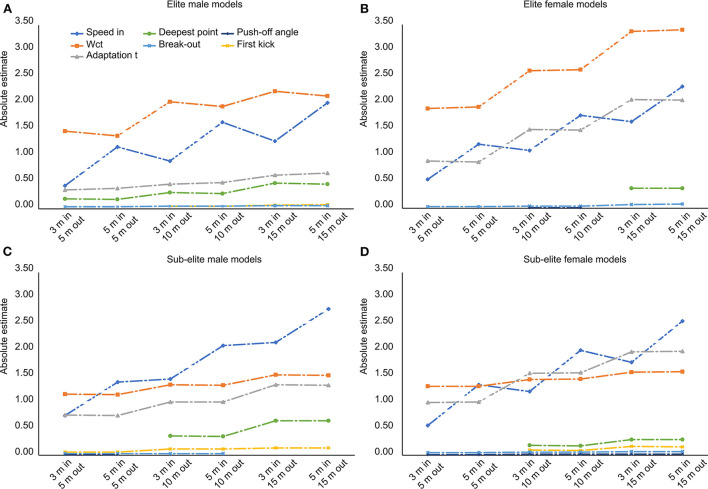
**(A–D)** The absolute estimates of the seven contributors (speed-in, adaptation time, wall contact time (WCT), break-out distance, deepest point in the underwater phase, distance at first kick and push-off angle) to the elite male and female and sub-elite male and female models are presented graphically. The different models are presented on the *x*-axis.

The estimates of the distance of the first kick had a negative sign for the sub-elite male swimmers, but a positive sign for the elite male and female swimmers and the sub-elite female swimmers. Compared across the different models, the contribution of the speed-in increased with the distance toward the wall covered in the performance measure (Figure 3, Appendix A). In contrast, the contribution of the adaptation time increased mainly for performance measures with an increased distance out-off the wall but decreased when the distance to the wall became longer.

The overall contribution of the WCT seemed higher for the elite swimmers than the sub-elite swimmers, for whom the speed-in was the predictor with the highest contribution to performance, especially for performance measures with long(er) distances. The adaptation time was a stronger performance predictor in the female swimmers than the male swimmers (Figure 3, Appendix A).

In the sub-elite female swimmers, all three main predictors (speed-in, adaptation time, and wall contact time) contributed to a similar degree, unlike the other three subgroups in which one main predictor clearly contributed more than the other two (notably WCT in the elite male and female swimmers and speed-in in the sub-elite male swimmers) (Figure 3, Appendix A).

## Discussion

The present study examined the implications of selecting a particular distance-based performance measure for the tumble turn with regard to the overall turn performance and the underlying performance determining variables. To this end, three statistical analyses were performed: (1) a foundational analysis to examine the impact of performance level and sex on the selected performance measures and the performance determining variables, (2) a coarse-grained analysis of the correlation strengths between the performance measures of interest, and (3) a fine-grained analysis of the technical variables that account for the variation in each of those performance measures.

### Foundational analysis

As expected, the foundational analysis revealed significant main effects of both performance level and sex on the performance measures and technical variables of interest. Based on these results the data set was divided into four subgroups: elite male, elite female, sub-elite male and sub-elite female. All subsequent analyses were done on the entire data set and on the four subgroups separately to gain insight into any subgroup-specific differences in performance and the adopted turn technique.

### Coarse-grained analysis

The level of association between the fixed-distance performance measures of interest was assessed by calculating repeated measures correlation coefficients for all possible pairs of performance measures, both for the entire set of turn data and the four subgroups. This analysis revealed a high overall level of association with correlation coefficients ranging from 0.84 to 0.99. Although this result was expected on logical grounds since the performance measures covering longer distances overlap with the performance measures covering smaller distances (e.g., 5 m-in−5 m-out is part of the 5 m-in−10 m-out and 5 m-in−15 m-out), it has, to our knowledge, not been established or highlighted before in the literature. The strong correlation between the various performance measures implies that if one is interested solely in assessing the overall turn performance, the choice of performance measures is less critical. This finding was exacerbated by the marginal unsystematic differences in the results obtained between the four subgroups, which showed similar correlation patterns independent of performance level and sex. The small differences in the level of association between the various performance measures may have been due to differences in the turn-related actions and amount of free-swimming covered by the different performance measures (e.g., the moment of head resurfacing and stroke resumption occurring somewhere between 5 and 15 m).

However, even though all correlation coefficients were higher than 0.8, it appeared that the included distance after the turn had a stronger effect than the distance before the turn since the correlation coefficient decreased pair-wise with different out-off-the-wall distances, but not with different to-the-wall distances (see Table 3). This finding can be interpreted by realizing that the speed after the turn is largely determined by an effective push-off, an appropriate time spent on the wall and a properly streamlined position (Welham and Thompson, 1997; Lyttle et al., 1998; Mason and Cossor, 2001) and that some of these variables may be affected by the characteristics of the approach toward the wall. The longer the distance covered, the more variability may be generated by the interaction of all performance determining factors. This might explain why the level of association diminishes faster when correlating performance measures with different out-of-the-wall distances compared to different into-the-wall distances. Therefore, performance measures with longer out-of-the-wall distances seem more informative about the efficiency of turning actions and the transfer to swimming than performance measures with shorter out-of-the-wall distances.

### Fine-grained analysis

The fine-grained sensitivity analysis revealed which turn-related actions are reflected predominantly in the performance measures of interest. This was achieved by finding the best fitting linear mixed-effects model for each performance measure using seven (eight minus one) technical variables as independent predictors and athlete ID as a random effect.

All the models showed a high *R*^2^ value ranging from 70 to 91% explained variance. This is in contrast to the findings of Nicol et al., who did not find any of the technical variables to be a significant predictor of the overall turn time (Nicol et al., 2019). This discrepancy might be explained by the size of the included data set and the variability that was introduced in the present study by including swimmers of different performance levels. Interestingly, all models starting 5 m from the wall showed a higher *R*^2^ than models starting 3 m before the wall. Speed-in, adaptation time, WCT, and break-out distance were significant predictors in all models. Of these four variables, speed-in was the only variable whose contribution differed between the 3 m-in and 5 m-in models. However, this is not surprising because the speed-in was measured between the 5 m and 3 m mark before the wall. The difference in contribution, therefore, seems to account for the higher explained variance of the 5 m-in models.

The estimates of speed-in had a negative sign, indicating that a higher swim speed toward the wall was associated with a better turn performance, as has been found in previous studies (Takahashi et al., 1982; Blanksby et al., 1996; Lyttle and Mason, 1997). The amount of variance explained by speed-in increased with increasing the distance covered by the performance measure. Taking an example from the elite male model (see Appendix A), an increase of speed-in of about 1 m/s would be associated with a turn time reduction of 0.44 s in the 3 m-in−5 m-out measure, while the same change would be associated with a turn time reduction of 2.32 s in the 5 m in−15 m out measure. This can be understood from the fact that the longer performance distances included more free-swimming; a higher swimming speed was, therefore, reflected more in the performance measures with longer distances. This could also explain why the contribution of the speed-in increased more strongly with distance in the sub-elite swimmers, who on average traveled a shorter distance underwater than the elite swimmers.

As expected, adaptation time and WCT turned out to be strong positive contributors to the overall turn performance. This finding is in agreement with the results of Blanksby and Puel (Blanksby et al., 1996; Puel et al., 2012), who also found that a reduction of the adaptation time and WCT resulted in faster turns. The adaptation time and WCT were reflected more strongly in the performance measures with shorter distances as indicated by their higher contribution to these models, which was expected as well. The model outcomes further revealed that the overall contribution of the WCT was higher for the elite compared to the sub-elite swimmers (Figure 3), which suggests that focusing the training on the optimization of WCT might have a beneficial effect on turn performance. However, as the WCT is very short (~0.3 s), easier improvements might be accomplished by shortening the adaptation time (~0.9 s). Another interesting finding of the fine-grained analysis was that the adaptation time is a stronger predictor in female compared to male swimmers, whereas the adaptation time itself was not significantly different between sexes. This suggests that a different turning technique was employed by male and female swimmers.

The contribution of the adaptation time and WCT decreased compared to the speed-in as the distance covered by the performance measures increased for all subgroups, except for the elite female swimmers. This decrease could be expected since these variables play a role early in the turn; therefore, even if their impact on turn time is large, it will be diluted by the impact of the actions later in the turn. This effect will be especially strong for the adaptation time. Also, the ratio of the WCT and the turn time decreases the longer the distance covered, which automatically reduces the impact. This result confirms our expectation that if the WCT is the focus of interest, a performance measure with short(er) distances should be chosen. The contradictory finding for the elite female swimmers could be explained by looking further into the characteristics of this subgroup, which contained a relatively small number of swimmers (*N* =15). This could have resulted in an overall higher variability of the included variables and an underestimation of their contribution.

The negative coefficient for the break-out distance is in line with the results of Mason et al., who concluded that a longer underwater phase is beneficial for the speed after the turn, resulting in a better turn performance (Mason and Cossor, 2001). The increase in the contribution of the break-out distance is also in line with our expectations because this variable plays out relatively late in the turn and thus was expected to contribute to the performance measures with longer distances. However, the contribution to the performance measures was generally much smaller than those of the speed-in and the WCT and also the relative impact declined with increasing distance when compared to the speed-in, which can be appreciated from the standardized estimates (see Appendix A).

By comparing the three main contributors (speed-in, adaptation time and WCT) between the subgroups, not one predictor appeared more dominant than the other two predictors in the sub-elite female swimmers. In contrast, WCT stood out as the main contributor in the elite male and female swimmers, while speed-in was the main contributor in the sub-elite male swimmers. This might suggest that the variation of turn strategies was markedly higher in the sub-elite female swimmers than in the other subgroups.

The remaining three technical variables, the push-off angle, the distance of the first kick and the deepest point in the underwater trajectory, were only minor contributors to some of the models. From the literature, a negative estimate was expected for the distance of the first kick (Lyttle et al., 2000). Surprisingly, this was only the case for the sub-elite male swimmers; in contrast, positive estimates of the distance of the first kick were found for the sub-elite male and female swimmers, while it did not even appear as a significant contributor in the elite female swimmers. Besides only playing a minor role to predict turn performance, the negative estimates of the distance of the first kick resulted from the relatively short distance to the wall of the sub-elite male swimmer (~1.82 m), while the elite male (~2.72 m) and sub-elite female swimmers (~2.55 m) might already had reached the optimal distance where a further increase would no longer be beneficial.

The deepest point in the underwater trajectory contributed to all elite male models, but only to the 10 m-out and longer sub-elite male models. Whereas the elite male swimmers reached a significantly greater depth during the underwater phase, their push-off angle was similar to that of their sub-elite counterparts. This suggests that the elite male swimmers held their downward line longer after push-off than the sub-elite male swimmers, which could also explain the later break-out distance. This is in line with the findings of Mason et al., who showed that swimmers with a longer underwater phase after the turn could benefit more than other swimmers from making quicker turns (Puel et al., 2010a).

Interestingly, the underwater phase seemed less important in the elite female swimmers, because none of the variables related to underwater swimming contributed to many of the derived models. This may be the case because the elite female swimmers within the data set adopted a wide variety of turn strategies, which could have obscured finding clear statistical relationships.

When comparing the subgroups with each other it became clear that there are some indications of different turning strategies between the subgroups. Furthermore, depending on the subgroup one is interested in, not all performance measures reflected the turn-related technical variables to the same degree. Therefore, it is important to choose a performance measure that reflects the variable of interest for the specific subgroup or question of interest. More research is needed to clarify these differences and their origins.

Although the standardized estimates enabled comparison, they should be interpreted with caution when comparing them between models. If the standard deviations between models are different, this could jeopardize drawing a valid conclusion based on their relative contributions. One example is the increase of standardized estimates for speed-in for the models for performance measures including a 3 m distance to the wall, while the standardized estimates decreased for models on the 5 m-in distances in elite swimmers. Using the non-standardized estimates showed that the effect of the speed-in variable increased with increasing distance from the wall, which is consistent with the idea that swimmers with a higher performance level have a higher speed-in and also will be faster after the turn.

One of the limitations of the present study is that the data set used contained no information about the underwater swimming ability of the swimmer, apart from the distance at which the head resurfaced after the turn. Although it was found in previous studies that underwater actions are important predictors of turn performance, this was not reflected in the present results (Clothier, 2004; Connaboy et al., 2016). It could be that the selected technical variables did not optimally reflect the underwater actions. The second limitation is that the data set only contained variables that were extractable from video footage. Adding biomechanical variables like the push-off force to the models would have rendered the analysis more comprehensive and should ideally be done in future research. However, most swimming facilities do not have the advanced measurement infrastructure required for this purpose, which precludes them from including other biomechanical variables besides kinematic variables.

The data set used in this study is unique as it contained over 2,800 turns from a broad variety of competitive swimmers, ranging from international Olympic finalists to regional swimmers. A sample size of this magnitude is unique in turn-related swimming research and allowed gaining a better understanding of the implications of choosing particular performance measures for both scientific and practical purposes. In particular, the present results clearly indicate that the various performance measures used in the literature are strongly correlated, they reflect different technical, performance determining variables, that play out differently in a subgroup-specific manner, depending on both performance level and sex. This implies that the choice for a particular performance measure has to be based on how well it is suited to answer the research or practical question of interest, and to which technical variables are reflected in the selected measure. The present results provide a wealth of information in this regard, which allows both researchers and coaches to make better-informed choices on which performance measures to use for their research or training purposes, and hopefully also inspires them to explore new leads in research and training.

### Conclusion

The present results suggest that if one is interested solely in the overall turn performance, the choice of performance measure is less critical and can be adapted in accordance with the available facilities or personal preferences of the coach or researcher. Even though all correlation coefficients were higher than 0.8, it has to be taken into account that the included distance after the turn was a stronger contributor than the distance before the turn. When the aim is to identify performance determining variables that could be improved through training, the choice of performance measure matters. The present results showed that choosing a fixed-distance performance measure with short distances reflects the WCT better while performance measures with long distances are beneficial if the focus is on the approach speed prior to the turn. Also, performance measures with short distances before the turn are more sensitive to changes in the adaptation time.

## Data availability statement

The data set presented in this article are not readily available because the data set contains elite athletes that can be identified from the data. Requests to access the data set should be directed to p.koster@fieldlabswimming.com.

## Ethics statement

The studies involving human participants were reviewed and approved by the Scientific and Ethical Review Board (VCWE) of the Faculty of Behavioural and Movement Sciences of the Vrije Universiteit Amststerdam. Written informed consent to participate in this study was provided by the participants' legal guardian/next of kin.

## Author contributions

PK, WA, SS, and PB conceptualized and designed the study. PK, WA, and SD performed the literature search and data processing. SS, PK, and WA were involved in data collection. PK, SD, SS, and PB drafted the current manuscript. All authors contributed substantially to the interpretation of the data, revised it critically and approved the final version of the manuscript before submission.

## Conflict of interest

The authors declare that the research was conducted in the absence of any commercial or financial relationships that could be construed as a potential conflict of interest.

## Publisher's note

All claims expressed in this article are solely those of the authors and do not necessarily represent those of their affiliated organizations, or those of the publisher, the editors and the reviewers. Any product that may be evaluated in this article, or claim that may be made by its manufacturer, is not guaranteed or endorsed by the publisher.
