# Supplementary material for: Implications of the choice of distance-based measures in assessing and investigating tumble turn performance
Source: Front Sports Act Living. 2022 Sep 22;4:958548. doi: 10.3389/fspor.2022.958548 (PMC9536243; doi:10.3389/fspor.2022.958548)
Supplement: Supplementary file 1 [file Data_Sheet_1.docx]

## Appendix

Appendix A. The table displays the intercepts, estimates (Est.), standard errors (S.E) and t-values for the different independent variables for each performance measure separately for the elite male and female and sub-elite male and female swimmers separately. An empty cell in the table implies that the corresponding value did not add significantly to the model fit. WCT stands for wall contact time.

| **Appendix A**  Elite male mixed effect models | | | | | | | | | | | | | | | | |  |  |
| --- | --- | --- | --- | --- | --- | --- | --- | --- | --- | --- | --- | --- | --- | --- | --- | --- | --- | --- |
|  | 3 m in - 5 m out | | | 5 m in - 5 m out | | | 3 m in - 10 m out | | | 5 m in - 10 m out | | | 3 m in - 15 m out | | | 5 m in - 15 m out | | |
|  | Est. | S.E. | t-value | Est. | S.E. | t-value | Est. | S.E. | t-value | Est. | S.E. | t-value | Est. | S.E. | t-value | Est. | S.E. | t-value |
| Intercept | 3.85 | 0.12 | 31.69 | 6.30 | 0.12 | 51.88 | 7.19 | 0.21 | 33.88 | 9.62 | 0.21 | 46.26 | 10.43 | 0.32 | 32.40 | 12.86 | 0.32 | 40.54 |
| Speed in | -0.44 | 0.04 | -10.41 | -1.18 | 0.04 | -27.58 | -0.91 | 0.07 | -12.69 | -1.65 | 0.07 | -23.30 | -1.29 | 0.11 | -11.80 | -2.02 | 0.11 | -18.78 |
| WCT | 1.48 | 0.11 | 13.50 | 1.39 | 0.11 | 12.63 | 2.04 | 0.19 | 10.98 | 1.95 | 0.18 | 10.67 | 2.24 | 0.28 | 7.97 | 2.15 | 0.28 | 7.74 |
| Adaptation t | 0.36 | 0.05 | 7.63 | 0.39 | 0.05 | 8.28 | 0.47 | 0.08 | 5.86 | 0.50 | 0.08 | 6.38 | 0.64 | 0.12 | 5.34 | 0.68 | 0.12 | 5.68 |
| First kick |  |  |  |  |  |  | 0.05 | 0.08 | 2.45 | 0.05 | 0.02 | 2.72 | 0.07 | 0.03 | 2.39 | 0.08 | 0.03 | 2.57 |
| Break-out | -0.04 | 0.00 | -7.71 | -0.04 | 0.00 | -7.21 | -0.05 | 0.01 | -5.91 | -0.05 | 0.01 | -5.69 | -0.06 | 0.01 | -5.10 | -0.06 | 0.01 | -4.96 |
| Push-off angle |  |  |  |  |  |  |  |  |  |  |  |  |  |  |  |  |  |  |
| Deepest point | 0.19 | 0.05 | 3.94 | 0.18 | 0.05 | 3.60 | 0.31 | 0.08 | 3.77 | 0.29 | 0.08 | 3.66 | 0.49 | 0.12 | 3.93 | 0.47 | 0.12 | 3.86 |
| R^2^ |  |  | 0.77 |  |  | 0.87 |  |  | 0.74 |  |  | 0.83 |  |  | 0.71 |  |  | 0.77 |
|  |  |  |  |  |  |  |  |  |  |  |  |  |  |  |  |  |  |  |
| Standardized elite male mixed effect models | | | | | | | | | | | | | | | | |  |  |
|  | 3 m in - 5 m out | | | 5 m in - 5 m out | | | 3 m in - 10 m out | | | 5 m in - 10 m out | | | 3 m in - 15 m out | | | 5 m in - 15 m out | | |
|  | Est. | S.E. | t-value | Est. | S.E. | t-value | Est. | S.E. | t-value | Est. | S.E. | t-value | Est. | S.E. | t-value | Est. | S.E. | t-value |
| Intercept |  |  |  |  |  |  |  |  |  |  |  |  |  |  |  |  |  |  |
| Speed in | -0.27 | 0.03 | -10.41 | -0.54 | 0.02 | -27.58 | -0.34 | 0.03 | -12.69 | -0.52 | 0.02 | -23.30 | -0.33 | 0.03 | -11.80 | -0.46 | 0.02 | -18.78 |
| WCT | 0.33 | 0.02 | 13.50 | 0.24 | 0.02 | 12.63 | 0.29 | 0.03 | 10.98 | 0.23 | 0.02 | 10.67 | 0.22 | 0.03 | 7.97 | 0.18 | 0.02 | 7.74 |
| Adaptation t | 0.16 | 0.02 | 7.63 | 0.13 | 0.02 | 8.28 | 0.13 | 0.02 | 5.86 | 0.11 | 0.02 | 6.38 | 0.12 | 0.02 | 5.34 | 0.11 | 0.02 | 5.68 |
| First kick |  |  |  |  |  |  | 0.05 | 0.02 | 2.45 | 0.05 | 0.02 | 2.72 | 0.05 | 0.02 | 2.39 | 0.05 | 0.02 | 2.57 |
| Break-out | -0.24 | 0.03 | -7.71 | -0.17 | 0.02 | -7.21 | -0.19 | 0.03 | -5.91 | -0.15 | 0.03 | -5.69 | -0.17 | 0.03 | -5.10 | -0.15 | 0.03 | -4.96 |
| Push-off angle |  |  |  |  |  |  |  |  |  |  |  |  |  |  |  |  |  |  |
| Deepest point | 0.09 | 0.02 | 3.94 | 0.07 | 0.02 | 3.60 | 0.10 | 0.03 | 3.77 | 0.08 | 0.02 | 3.66 | 0.10 | 0.03 | 3.93 | 0.09 | 0.02 | 3.86 |
| R^2^ |  |  | 0.77 |  |  | 0.87 |  |  | 0.74 |  |  | 0.83 |  |  | 0.71 |  |  | 0.77 |

| Sub-elite male mixed effect models | | | | | | | | | | | | | | | | |  |  |
| --- | --- | --- | --- | --- | --- | --- | --- | --- | --- | --- | --- | --- | --- | --- | --- | --- | --- | --- |
|  | 3 m in - 5 m out | | | 5 m in - 5 m out | | | 3 m in - 10 m out | | | 5 m in - 10 m out | | | 3 m in - 15 m out | | | 5 m in - 15 m out | | |
|  | Est. | S.E. | t-value | Est. | S.E. | t-value | Est. | S.E. | t-value | Est. | S.E. | t-value | Est. | S.E. | t-value | Est. | S.E. | t-value |
| Intercept | 4.63 | 0.16 | 29.67 | 6.90 | 0.16 | 43.63 | 8.18 | 0.28 | 29.41 | 10.45 | 0.28 | 37.37 | 12.18 | 0.39 | 31.17 | 14.45 | 0.39 | 36.82 |
| Speed in | -0.76 | 0.06 | -12.00 | -1.40 | 0.06 | -21.78 | -1.46 | 0.11 | -13.20 | -2.10 | 0.11 | -18.84 | -2.16 | 0.15 | -14.03 | -2.80 | 0.15 | -18.08 |
| WCT | 1.17 | 0.07 | 17.46 | 1.16 | 0.07 | 17.11 | 1.35 | 0.12 | 11.69 | 1.34 | 0.12 | 11.55 | 1.54 | 0.16 | 9.50 | 1.53 | 0.16 | 9.40 |
| Adaptation t | 0.77 | 0.07 | 11.43 | 0.76 | 0.07 | 11.16 | 1.02 | 0.11 | 9.13 | 1.02 | 0.11 | 9.02 | 1.35 | 0.16 | 8.50 | 1.34 | 0.16 | 8.41 |
| First kick | -0.06 | 0.02 | -3.64 | -0.06 | 0.02 | -3.60 | -0.12 | 0.03 | -3.97 | -0.12 | 0.03 | -3.95 | -0.14 | 0.04 | -3.29 | -0.14 | 0.04 | -3.27 |
| Break-out | -0.03 | 0.01 | -4.83 | -0.03 | 0.01 | -4.61 | 0.03 | 0.01 | 3.05 | 0.03 | 0.01 | 3.12 |  |  |  |  |  |  |
| Push-off angle | 0.01 | 0.00 | 3.76 | 0.01 | 0.00 | 3.64 |  |  |  |  |  |  |  |  |  |  |  |  |
| Deepest point |  |  |  |  |  |  | 0.37 | 0.10 | 3.70 | 0.36 | 0.10 | 3.65 | 0.66 | 0.12 | 5.67 | 0.66 | 0.12 | 5.66 |
| R^2^ |  |  | 0.88 |  |  | 0.91 |  |  | 0.88 |  |  | 0.90 |  |  | 0.86 |  |  | 0.88 |
|  |  |  |  |  |  |  |  |  |  |  |  |  |  |  |  |  |  |  |
| Standardized sub-elite male mixed effect models | | | | | | | | | | | | | | | | |  |  |
|  | 3 m in - 5 m out | | | 5 m in - 5 m out | | | 3 m in - 10 m out | | | 5 m in - 10 m out | | | 3 m in - 15 m out | | | 5 m in - 15 m out | | |
|  | Est. | S.E. | t-value | Est. | S.E. | t-value | Est. | S.E. | t-value | Est. | S.E. | t-value | Est. | S.E. | t-value | Est. | S.E. | t-value |
| Intercept |  |  |  |  |  |  |  |  |  |  |  |  |  |  |  |  |  |  |
| Speed in | -0.27 | 0.02 | -12.00 | -0.41 | 0.02 | -21.78 | -0.30 | 0.02 | -13.20 | -0.39 | 0.02 | -18.84 | -0.32 | 0.02 | -14.03 | -0.38 | 0.02 | -18.08 |
| WCT | 0.25 | 0.01 | 17.46 | 0.20 | 0.01 | 17.11 | 0.17 | 0.01 | 11.69 | 0.15 | 0.01 | 11.55 | 0.14 | 0.01 | 9.50 | 0.13 | 0.01 | 9.40 |
| Adaptation t | 0.20 | 0.02 | 11.43 | 0.16 | 0.01 | 11.16 | 0.15 | 0.02 | 9.13 | 0.14 | 0.02 | 9.02 | 0.15 | 0.02 | 8.50 | 0.13 | 0.02 | 8.41 |
| First kick | -0.06 | 0.02 | -3.64 | -0.05 | 0.01 | -3.60 | -0.06 | 0.02 | -3.97 | -0.05 | 0.01 | -3.95 | -0.05 | 0.02 | -3.29 | -0.05 | 0.01 | -3.27 |
| Break-out | -0.09 | 0.02 | -4.83 | -0.08 | 0.02 | -4.61 | 0.07 | 0.02 | 3.05 | 0.06 | 0.02 | 3.12 |  |  |  |  |  |  |
| Push-off angle | 0.06 | 0.02 | 3.76 | 0.05 | 0.01 | 3.64 |  |  |  |  |  |  |  |  |  |  |  |  |
| Deepest point |  |  |  |  |  |  | 0.07 | 0.02 | 3.70 | 0.06 | 0.02 | 3.65 | 0.09 | 0.02 | 5.67 | 0.09 | 0.02 | 5.66 |
| R^2^ |  |  | 0.88 |  |  | 0.91 |  |  | 0.88 |  |  | 0.90 |  |  | 0.86 |  |  | 0.88 |

| Elite female mixed effect models | | | | | | | | | | | | | | | | |  |  |
| --- | --- | --- | --- | --- | --- | --- | --- | --- | --- | --- | --- | --- | --- | --- | --- | --- | --- | --- |
|  | 3 m in - 5 m out | | | 5 m in - 5 m out | | | 3 m in - 10 m out | | | 5 m in - 10 m out | | | 3 m in - 15 m out | | | 5 m in - 15 m out | | |
|  | Est. | S.E. | t-value | Est. | S.E. | t-value | Est. | S.E. | t-value | Est. | S.E. | t-value | Est. | S.E. | t-value | Est. | S.E. | t-value |
| Intercept | 3.91 | 0.14 | 27.27 | 6.23 | 0.14 | 43.16 | 7.26 | 0.24 | 29.93 | 9.59 | 0.24 | 39.41 | 10.63 | 0.39 | 27.02 | 12.95 | 0.39 | 32.92 |
| Speed in | -0.55 | 0.05 | -11.15 | -1.22 | 0.05 | -24.36 | -1.10 | 0.08 | -13.10 | -1.77 | 0.08 | -20.94 | -1.65 | 0.14 | -11.99 | -2.32 | 0.14 | -16.81 |
| WCT | 1.90 | 0.11 | 17.98 | 1.93 | 0.11 | 18.03 | 2.62 | 0.18 | 14.67 | 2.64 | 0.18 | 14.75 | 3.37 | 0.29 | 11.48 | 3.40 | 0.29 | 11.56 |
| Adaptation t | 0.90 | 0.08 | 11.30 | 0.88 | 0.08 | 11.04 | 1.50 | 0.14 | 10.99 | 1.49 | 0.14 | 10.86 | 2.07 | 0.22 | 9.51 | 2.06 | 0.22 | 9.45 |
| First kick |  |  |  |  |  |  |  |  |  |  |  |  |  |  |  |  |  |  |
| Break-out | -0.03 | 0.00 | -7.36 | -0.03 | 0.00 | -7.64 | -0.04 | 0.01 | -5.33 | -0.04 | 0.01 | -5.44 | -0.07 | 0.01 | -5.66 | -0.08 | 0.01 | -5.73 |
| Push-off angle |  |  |  |  |  |  | 0.01 | 0.00 | 2.24 | 0.01 | 0.00 | 2.15 |  |  |  |  |  |  |
| Deepest point |  |  |  |  |  |  |  |  |  |  |  |  | 0.38 | 0.12 | 3.21 | 0.38 | 0.12 | 3.17 |
| R^2^ |  |  | 0.79 |  |  | 0.86 |  |  | 0.76 |  |  | 0.81 |  |  | 0.7 |  |  | 0.75 |
|  |  |  |  |  |  |  |  |  |  |  |  |  |  |  |  |  |  |  |
| Standardized elite female mixed effect models | | | | | | | | | | | | | | | | |  |  |
|  | 3 m in - 5 m out | | | 5 m in - 5 m out | | | 3 m in - 10 m out | | | 5 m in - 10 m out | | | 3 m in - 15 m out | | | 5 m in - 15 m out | | |
|  | Est. | S.E. | t-value | Est. | S.E. | t-value | Est. | S.E. | t-value | Est. | S.E. | t-value | Est. | S.E. | t-value | Est. | S.E. | t-value |
| Intercept |  |  |  |  |  |  |  |  |  |  |  |  |  |  |  |  |  |  |
| Speed in | -0.27 | 0.02 | -11.15 | -0.48 | 0.02 | -24.36 | -0.35 | 0.03 | -13.10 | -0.48 | 0.02 | -20.94 | -0.37 | 0.03 | -11.99 | -0.47 | 0.03 | -16.81 |
| WCT | 0.45 | 0.02 | 17.98 | 0.37 | 0.02 | 18.03 | 0.40 | 0.03 | 14.67 | 0.35 | 0.02 | 14.75 | 0.36 | 0.03 | 11.48 | 0.33 | 0.03 | 11.56 |
| Adaptation t | 0.29 | 0.03 | 11.30 | 0.23 | 0.02 | 11.04 | 0.31 | 0.03 | 10.99 | 0.27 | 0.02 | 10.86 | 0.31 | 0.03 | 9.51 | 0.28 | 0.03 | 9.45 |
| First kick |  |  |  |  |  |  |  |  |  |  |  |  |  |  |  |  |  |  |
| Break-out | -0.20 | 0.03 | -7.36 | -0.17 | 0.02 | -7.64 | -0.19 | 0.03 | -5.33 | -0.17 | 0.03 | -5.44 | -0.23 | 0.04 | -5.66 | -0.21 | 0.04 | -5.73 |
| Push-off angle |  |  |  |  |  |  | 0.08 | 0.03 | 2.24 | 0.06 | 0.03 | 2.15 |  |  |  |  |  |  |
| Deepest point |  |  |  |  |  |  |  |  |  |  |  |  | 0.11 | 0.04 | 3.21 | 0.09 | 0.03 | 3.17 |
| R^2^ |  |  | 0.79 |  |  | 0.86 |  |  | 0.76 |  |  | 0.81 |  |  | 0.7 |  |  | 0.75 |

| Sub-elite female mixed effect models | | | | | | | | | | | | | | | | |  |  |
| --- | --- | --- | --- | --- | --- | --- | --- | --- | --- | --- | --- | --- | --- | --- | --- | --- | --- | --- |
|  | 3 m in - 5 m out | | | 5 m in - 5 m out | | | 3 m in - 10 m out | | | 5 m in - 10 m out | | | 3 m in - 15 m out | | | 5 m in - 15 m out | | |
|  | Est. | S.E. | t-value | Est. | S.E. | t-value | Est. | S.E. | t-value | Est. | S.E. | t-value | Est. | S.E. | t-value | Est. | S.E. | t-value |
| Intercept | 4.27 | 0.13 | 31.41 | 6.76 | 0.13 | 53.77 | 7.74 | 0.22 | 34.76 | 10.24 | 0.22 | 44.39 | 11.39 | 0.32 | 35.98 | 13.89 | 0.31 | 44.15 |
| Speed in | -0.57 | 0.05 | -10.48 | -1.35 | 0.05 | -25.34 | -1.22 | 0.09 | -13.57 | -2.01 | 0.09 | -22.55 | -1.78 | 0.13 | -13.95 | -2.57 | 0.13 | -20.25 |
| WCT | 1.32 | 0.05 | 25.22 | 1.32 | 0.05 | 25.59 | 1.45 | 0.09 | 15.39 | 1.46 | 0.09 | 15.65 | 1.59 | 0.13 | 11.86 | 1.60 | 0.13 | 12.02 |
| Adaptation t | 1.01 | 0.06 | 18.09 | 1.02 | 0.06 | 18.49 | 1.57 | 0.09 | 16.99 | 1.58 | 0.09 | 17.26 | 1.98 | 0.13 | 15.02 | 1.99 | 0.13 | 15.19 |
| First kick |  |  |  |  |  |  | 0.10 | 0.02 | 3.98 | 0.09 | 0.02 | 3.81 | 0.17 | 0.04 | 4.68 | 0.16 | 0.04 | 4.57 |
| Break-out | -0.05 | 0.01 | -10.04 | -0.05 | 0.01 | -9.64 | -0.06 | 0.01 | -6.33 | -0.06 | 0.01 | -6.05 | -0.07 | 0.01 | -5.61 | -0.07 | 0.01 | -5.41 |
| Push-off angle | 0.01 | 0.00 | 5.70 | 0.01 | 0.00 | 5.83 | 0.02 | 0.00 | 5.05 | 0.02 | 0.00 | 5.14 | 0.02 | 0.00 | 4.05 | 0.02 | 0.00 | 4.10 |
| Deepest point |  |  |  |  |  |  | 0.19 | 0.09 | 2.12 | 0.18 | 0.09 | 2.08 | 0.30 | 0.13 | 2.39 | 0.30 | 0.13 | 2.36 |
| R^2^ |  |  | 0.85 |  |  | 0.90 |  |  | 0.85 |  |  | 0.89 |  |  | 0.85 |  |  | 0.87 |
|  |  |  |  |  |  |  |  |  |  |  |  |  |  |  |  |  |  |  |
| Standardized sub-elite female mixed effect models | | | | | | | | | | | | | | | | |  |  |
|  | 3 m in - 5 m out | | | 5 m in - 5 m out | | | 3 m in - 10 m out | | | 5 m in - 10 m out | | | 3 m in - 15 m out | | | 5 m in - 15 m out | | |
|  | Est. | S.E. | t-value | Est. | S.E. | t-value | Est. | S.E. | t-value | Est. | S.E. | t-value | Est. | S.E. | t-value | Est. | S.E. | t-value |
| Intercept |  |  |  |  |  |  |  |  |  |  |  |  |  |  |  |  |  |  |
| Speed in | -0.21 | 0.02 | -10.48 | -0.40 | 0.02 | -25.34 | -0.26 | 0.02 | -13.57 | -0.38 | 0.02 | -22.55 | -0.27 | 0.02 | -13.95 | -0.35 | 0.02 | -20.25 |
| WCT | 0.33 | 0.01 | 25.22 | 0.27 | 0.01 | 25.59 | 0.21 | 0.01 | 15.39 | 0.19 | 0.01 | 15.65 | 0.16 | 0.01 | 11.86 | 0.15 | 0.01 | 12.02 |
| Adaptation t | 0.30 | 0.02 | 18.09 | 0.25 | 0.01 | 18.49 | 0.27 | 0.02 | 16.99 | 0.24 | 0.01 | 17.26 | 0.24 | 0.02 | 15.02 | 0.22 | 0.01 | 15.19 |
| First kick |  |  |  |  |  |  | 0.06 | 0.02 | 3.98 | 0.05 | 0.01 | 3.81 | 0.07 | 0.02 | 4.68 | 0.06 | 0.01 | 4.57 |
| Break-out | -0.18 | 0.02 | -10.04 | -0.14 | 0.01 | -9.64 | -0.11 | 0.02 | -6.33 | -0.09 | 0.02 | -6.05 | -0.10 | 0.02 | -5.61 | -0.09 | 0.02 | -5.41 |
| Push-off angle | 0.10 | 0.02 | 5.70 | 0.08 | 0.01 | 5.83 | 0.09 | 0.02 | 5.05 | 0.08 | 0.02 | 5.14 | 0.07 | 0.02 | 4.05 | 0.07 | 0.02 | 4.10 |
| Deepest point |  |  |  |  |  |  | 0.04 | 0.02 | 2.12 | 0.03 | 0.02 | 2.08 | 0.05 | 0.02 | 2.39 | 0.04 | 0.02 | 2.36 |
| R^2^ |  |  | 0.84 |  |  | 0.9 |  |  | 0.85 |  |  | 0.89 |  |  | 0.85 |  |  | 0.87 |
